# Calcium propionate in tortillas – a likely cause of a large outbreak of acute gastrointestinal illness, Finland, 2023

**DOI:** 10.2807/1560-7917.ES.2026.31.11.2600185

**Published:** 2026-03-19

**Authors:** Veera Varo, Elina Leinonen, Annukka Markkula, Minna Anthoni, Marja Raatikainen, Maria Tirkkonen, Nina Gynther, Heidi Landgren, Merja Korkalainen, Ruska Rimhanen-Finne

**Affiliations:** 1 Department of Public Health, Institute for Health and Welfare, Helsinki, Finland; 2 Microbiological Food Safety Unit, Finnish Food Authority, Helsinki, Finland; 3 Chemical Food Safety Unit, Finnish Food Authority, Helsinki, Finland; 4 Chemistry Unit, Finnish Food Authority, Helsinki, Finland; 5 Environmental services of the Mikkeli region, Mikkeli, Finland; 6 Department of Veterinary Biosciences, University of Helsinki, Helsinki, Finland

**Keywords:** calcium propionate, outbreak, school lunch, chemical, tortilla

## Abstract

In August 2023, 721 individuals became ill with gastrointestinal symptoms in 17 schools in a Finnish municipality. Of these, 323 (45%) developed quickly passing symptoms during school lunch or < 30 min after the lunch. In a questionnaire-based retrospective cohort study, consumption of flour tortillas and vegetable filling served at lunch were statistically associated with illness (adjusted odds ratio (aOR) = 3.3; 95% confidence interval (CI): 1.4–7.4 for the tortillas and aOR = 1.5; 95% CI: 1.1–2.1 for the filling). Abnormal odour was observed in five of nine tortilla samples. In three samples from tortillas produced during a limited production period, high concentrations of calcium propionate (E 282) were measured (> 24,000 mg/kg), exceeding the regulatory maximum limit of 2,000 mg/kg. The tortillas served at lunch were traced to a manufacturer in another EU country. The manufacturer was unable to identify any cause in the production process that could explain the high concentrations of calcium propionate. Our results are in line with findings from other investigations that excessive calcium propionate can induce gastrointestinal symptoms. The investigation highlights the need for strengthened surveillance of chemical-related food-borne outbreaks in Europe and timely communication between school staff and public health authorities to support rapid outbreak detection and response.

**Key public health message**

**What did you want to address in this study and why?**

In 2023, a large food-borne outbreak linked to school lunches occurred in a Finnish municipality. We aimed to investigate the cause and the source of the outbreak and initiate measures to prevent similar events in the future.

**What have we learnt from this study?**

The likely cause of this outbreak was high concentration (> 24,000 mg/kg) of food preservative calcium propionate in flour tortillas served at school lunch. The concentration of the additive exceeded the permitted level (2,000 mg/kg). This study also highlights the importance of rapid communication between schools and public health authorities in controlling outbreaks.

**What are the implications of your findings for public health?**

Our findings remind us of chemical agents as causes of food-borne outbreaks and the need for enhanced surveillance in Europe. Schools should be better prepared for sudden-onset food-borne outbreaks, and guidelines to interrupt school meals for safety reasons should exist.

## Background

Chemicals can enter food either unintentionally or intentionally. Unintentional contamination may occur through environmental pollutants, processing, packaging or raw materials [1-4]. Chemical reactions during processing may also produce harmful by-products [1,3]. Additionally, cleaning agents, detergents and sanitisers used in food production may contribute to contamination of food products [3,4]. Natural toxins in plants, such as lectins in beans, may also pose health risks [5]. Residues from legally used agricultural chemicals and medicines may remain in food, and food spoilage can lead to the formation of toxic mycotoxins [6]. The European Union (EU) legislation sets maximum levels for contaminants and residues to ensure food safety [7,8].

In contrast, food additives are intentionally added to food to improve shelf life, flavour, texture or appearance [1,9]. Over 300 additives are authorised for use within the EU, each with specific conditions for use and maximum permitted concentrations as defined by the legislation [10,11].

Health effects from chemical contaminants are most often considered chronic, resulting from long-term, low-level exposure [2,12]. However, certain chemicals can cause acute illness with rapid symptom onset, depending on the amount or concentration of exposure [13]. Compared with biological agents, food-borne outbreaks caused by chemicals are less frequently reported [14]. Moreover, comprehensive EU data are lacking, as reporting of food-borne outbreaks to European Food Safety Authority (EFSA) does not include outbreaks caused by most chemical agents, except for biotoxins and microbial biogenic amines [15]. In Finland, food-borne and waterborne outbreaks caused by biological and chemical agents are reported by municipal authorities to the national Food- and Waterborne Outbreak (FWO) Registry.

Finland has a school meal programme, offering a daily school lunch free of charge to all students from preschool to upper secondary schools [16]. Participation in the school lunch is highest among younger students (aged 7–12 years), while older students eat the offered school lunch less frequently. School staff are allowed to purchase the school lunch. In many municipalities, meals are prepared in a central kitchen and distributed to satellite kitchens in schools.

## Outbreak detection

On Wednesday of 16 August 2023, a municipal environmental health authority (MEHA) in Municipality A in Eastern Finland received information from the central kitchen of ca 10–20 students falling ill with vomiting and nausea within 10 min after school lunch at five of the municipality’s 19 schools. On the same day, the MEHA notified the suspected food-borne outbreak to the FWO Registry (Figure). The next day, the MEHA created an online survey, and more than 100 persons reported gastrointestinal symptoms. The Finnish Institute for Health and Welfare (THL) and the Finnish Food Authority (FFA) joined the outbreak investigation to support and coordinate it.

**Figure:**
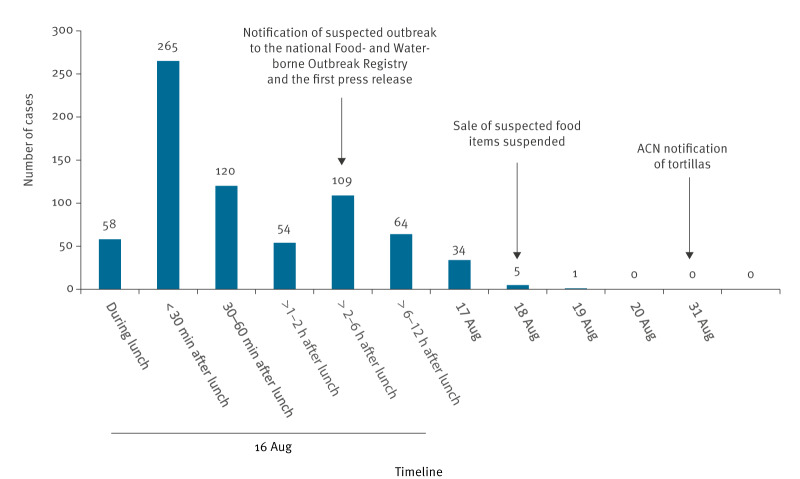
Timeline of symptom onset and outbreak control measures in an outbreak linked to consumption of tortillas at a school lunch, Eastern Finland, 16–31 August 2023

ACN:  The Alert and Cooperation Network (https://food.ec.europa.eu/food-safety/acn_en).

Here we report the investigation of the food-borne outbreak linked to consumption of food contaminated with food preservative calcium propionate and describe previous food-borne outbreaks caused by chemical agents reported to the FWO Registry between 2010 and 2022.

## Methods

### Outbreak case definition and case finding

On 17 August, the MEHA sent a link to an online questionnaire to the parents of students and staff of all schools of Municipality A. On 23 August, the link was also sent to the parents and staff of two preschool groups that had lunch at two schools. In the questionnaire, we asked about symptoms (diarrhoea, vomiting, stomach pain, nausea, fever (≥ 38°C), headache, muscle pain, joint pain, melaena), the students’ year group and exposure to each food item served.

We defined a case as a person who had lunch prepared with the same ingredients at one of schools of Municipality A on 16 August 2023 and reported vomiting, diarrhoea or stomach pain between 16 and 20 August 2023.

### Cohort study

We conducted a cohort study to investigate if the foods served at the schools were associated with symptoms. We included the following exposures based on the foods and beverages served: flour tortillas, vegetable filling (frozen Quorn mince, canned red kidney beans, canned tomato paste, frozen paprika strips, frozen onion cubes, sweet chili sauce, starch, spices), iceberg lettuce, tomato, canned pickled red onion, crispbread, margarine, milk and water. Respondents could answer ‘Yes’, ‘No’ or ‘I don’t know’. We included open-ended questions to gather information if the respondents had noticed anything out of the ordinary with the food.

### Traceback and food handling investigation

The MEHA collected information about lunch preparation and the traceability of the raw materials by interviewing staff responsible for serving food and receiving documents from them. The MEHA inspected the central kitchen on 17 August and 11 of the 14 school kitchen premises between 17 and 24 August. Three kitchens were contacted by phone. The inspectors collected food samples from the kitchens and checked the food safety management records such as temperature records.

The traceback investigation was conducted by the MEHA and the FFA, and they were in contact with other municipalities regarding food traceability by phone, email and remote meetings. The FFA was responsible for the international traceback investigation.

### Microbiological and chemical analyses of food samples

In total, nine samples from flour tortillas and eight from the vegetable filling or its components were analysed for staphylococcal enterotoxins (ISO 19020:2017) and cereulide (ISO 18465:2017), eight samples from tortillas and five from filling components for coagulase-positive staphylococci (NMKL 66:2009, modified) and *Bacillus cereus* (NMKL 67:2021), respectively. Samples from eight tortillas were also analysed for aerobic bacteria (NMKL 189:2017), Enterobacterales (NMKL 144:2005) and moulds (NMKL 98:2005, modified), and seven tortilla samples for aerobic and anaerobic sporulating bacteria (NMKL 189:2017), mycotoxins (liquid chromatography–mass spectrometry (LC-MS/MS) analysis, internal method), trace metals and phosphorus. Calcium concentrations were measured in nine tortilla samples. In addition, five tortilla samples were analysed for propionic acid (gas chromatography flame ionisation detector (GC-FID)), and the result was also calculated as calcium propionate. One sample from canned beans was analysed for lectin (immunoagglutination). All samples were analysed in official laboratories. The pH was tested on 11 tortilla samples. Nine flour tortilla samples were inspected for smell by local food inspectors or laboratory staff members after the packages were opened. More details of the methods are described in Supplementary Table.

### Data analysis

Sixteen of the 19 schools of the municipality had the same lunch menu. From the analysis, we excluded persons who did not have the school lunch (n = 207), persons reporting symptoms prior to the meal (n = 26) and students who had not answered the question about their year group (n = 5). Persons reporting having eaten gluten-free tortillas (n = 18) were coded as not having eaten flour tortillas.

We calculated food-specific attack rates (AR), relative risks (RR) and 95% confidence intervals (CI) for the food exposures served at schools. Exposures with a p value < 0.2 in the univariable analysis were included in the multivariable model. We used multivariable logistic regression, with clustering for schools to control for the potential confounding of the mutual effects of the food exposures, yielding adjusted odds ratios (aOR) and 95% CIs. The level of statistical significance was set to 0.01. The analyses were performed using Stata version 18.0 (Stata Corporation, the United States (US)).

### Food- and waterborne outbreaks in the Food- and Waterborne Outbreak Registry

In Finland, the THL and the FFA are responsible for jointly maintaining the FWO Registry. Since 2010, the reporting system has been digital with direct outbreak notifications from the municipal outbreak investigation groups (MOIGs), enabling real-time consultation by the national authorities. Once the MOIG has completed an investigation, reporting of an outbreak is mandatory when at least two persons have fallen ill with similar symptoms after consuming the same food or water [17].

Using the FWO Registry, we analysed the number of reported outbreaks linked to chemical agents and persons fallen ill in these outbreaks in 2010–2022.

## Results

### Descriptive epidemiology

The questionnaire was open between 17 and 23 August 2023. In the municipality, there were altogether 6,123 students and staff in the 19 schools. In total, 2,938 (54%) of the 5,430 students and 252 (36%) of the 693 staff responded (Table 1). Of the 3,190 respondents, 721 (23%) met the case definition. Of these, 95% (683/721) were students.

**Table 1:** Number of students and staff, response rate, attack rate and number of cases per group in a food-borne outbreak linked to consumption of tortillas at a school lunch, Eastern Finland, August 2023 (n = 6,123)^a^

Students and staff	Students and staff (n)	Respondents		Cases (n)	AE among respondents (%)	Proportion of cases per group (%)
		n	%			
Students						
Preschools and primary schools (age: 6–8 years)	1,004	664	66	143	22	14
Primary schools (age: 9–12 years)	2,078	1,294	62	384	30	18
Middle schools (age: 13–15 years)	1,613	768	48	92	12	6
Secondary schools (age: 16–19 years)	735	212	29	64	30	9
Total (students)	5,430	2,938	54	683	23	13
Staff						
Staff	693	252	36	38	38	5
Total (students and staff)	6,123	3,190	52	721	23	12

AE: attack rate.

^a^ The table presents the number of students and staff of the 19 schools of Municipality A, Eastern Finland.

The most common symptoms among the 721 cases were abdominal pain (n = 693; 96%), nausea (n = 452; 63%) and headache (n = 241; 33%), followed by diarrhoea (n = 119; 17%), vomiting (n = 84; 12%) and muscle pain (n = 60; 8%).

The number of cases peaked on 16 August, suggesting a sudden onset and a point source (Figure). Fifty-eight (8%) cases developed symptoms during lunch and 265 (37%) < 30 min after lunch. The median duration of illness was 4–12 h (range: < 2–57 h). Eighty-two (11%) cases had still symptoms when answering the questionnaire. No cases visited healthcare and there were no deaths.

### Traceback and food handling investigation

On 16 August 2023, 17 of 18 school kitchens in the municipality served tortillas with hot vegetable filling and salad. One school did not have a kitchen at the time of the outbreak. The students from that school had lunch at another school or outside the schools. Three schools had on-site kitchens and 14 had satellite kitchens supplied by the central kitchen. The vegetable filling was prepared in the three on-site kitchens and in the central kitchen, from which the filling was delivered hot to the satellite kitchens. The central kitchen also supplied meals to nurseries and preschools, but the outbreak day all except the groups that ate at the schools had different food.

At one school with an on-site kitchen, the tortillas and the filling came from other manufacturers. Persons who ate at this school were coded as ‘not eaten’. The remaining 16 schools served identical menus with ingredients from the same manufacturers. The tortillas were delivered packaged in a modified atmosphere (18 tortillas per package), opened at school kitchens and served unheated as instructed by the manufacturer. Diners filled their tortillas themselves.

The tortillas served at these 16 schools were of two batch numbers with the same best-before date. The tortillas were manufactured in an EU country A and shipped to Finland through an operator in an EU country B. Tortillas of the two batches were distributed to several places in Finland, including providers of community meals, but no gastrointestinal illnesses were reported in these localities, and no samples were taken. We do not have information on potential tortilla deliveries to other countries, but to our knowledge, the supplier had not received any additional customer feedback.

At a school meal in another municipality, tortillas packaged at 13:12 were served without any complaints. The same tortilla batches were also traced to four restaurants in Municipality A, and the MEHA collected tortilla samples from these restaurants. Three of these restaurants had not served the tortillas.

Ingredients of the vegetable filling, except for one of the spices and a thickener, were delivered across the country. Salad ingredients, which were also delivered to several other locations, were delivered to school kitchens in unopened packages.

The inspections of the central kitchen and the on-site and satellite kitchens did not reveal any hygiene breaches that could have explained the outbreak. In one school kitchen, the staff said that some tortillas were mouldy and these were discarded. The central kitchen and the schools had kept retention samples of served food according to the instructions of the MEHA. The health inspectors collected samples of vegetable fillings, salad and tortillas from opened and unopened packages.

### Results of microbiological and chemical analyses

Concentrations of calcium in seven of the nine tested flour tortilla samples were measured to 5,400–5,500 mg/kg, i.e. above the reference value of 810 mg/kg (Table 2). In three tortilla samples, concentrations of calcium propionate and propionic acid exceeded the maximum permitted level (MPL) of tortillas (2,000 mg/kg) (calcium propionate: 24,283–24,730 mg/kg; propionic acid: 19,320–19,675 mg/kg) [18]. Samples with elevated calcium, calcium propionate and propionic acid concentrations were from tortillas packaged between 13:24 and 13:32. In five tortilla samples, a vinegar-like odour was observed. Three of these were from tortillas packaged between 13:24 and 13:29, while the packaging time of the remaining two samples is unknown. No specific odour was observed in four samples from tortillas packaged between 16:33 and 21:17, and the calcium, calcium propionate and propionic acid concentrations were within normal range in two of these samples tested. The pH of the tortilla samples ranged from 5.4 to 5.7. According to the manufacturer, the target pH of tortillas was 5.5 (range: 5–6). Results of the other tests were either below the detection limits or within the normal range.

**Table 2:** Results of chemical and sensory analyses of tortilla samples (n = 15) in an investigation of an outbreak linked to consumption of tortillas at a school lunch, Eastern Finland, 2023

Sample ID^a^	Batch	Packaging time	Sampling site	Odour	pH^b^	Calcium (mg/kg)^c^	Calcium propionate (mg/kg)^d^	Propionic acid (mg/kg)
1	Not available	Not available	Primary school 1	Vinegar-like smell	5.7	NA	NA	NA
2	Not available	Not available		Vinegar-like smell	5.7	NA	NA	NA
3	Batch 1	13:24	Restaurant 1	Vinegar-like smell	NA	NA	NA	NA
4		13:24		NA	5.7	5,400	24,730	19,675 (± 2,233)
5	Batch 1	13:25	Combined primary and middle school 1	NA	5.7	5,400	NA	NA
6	Batch 1	13:26	Restaurant 2	NA	5.7	5,500	NA	NA
7		13:27		Vinegar-like smell	NA	NA	NA	NA
8		13:27		NA	5.7	5,500	NA	NA
9	Batch 1	13:28	Combined primary and middle school 1	NA	5.7	5,400	NA	NA
10		13:29		Vinegar-like smell	5.7	5,400	24,283	19,320 (± 2,198)
11	Batch 1	13:32	Primary school 2	NA	5.7	5,500	24,423	19,431 (± 2,209)
12	Batch 1	16:33	Secondary school	Without remark	5.4	580	1,944	1,547 (± 257)
13	Batch 1	16:48	Restaurant 3	Without remark	NA	NA	NA	NA
14	Batch 2	21:12	Combined primary and middle school 2	Without remark	NA	NA	NA	NA
15	Batch 2	21:17	Combined primary and middle school 3	Without remark	5.5	600	2,032	1,617 (± 267)

ID: identification code; NA: not analysed.

^a^ Sample 1 and 2 were frozen retention samples, samples 3-15 from unopened packages, sample 11 frozen.

^b^ Target pH according to the manufacturer:  5.5 (range: 5–6).

^c^ Reference value for tortillas according to Finland's National Food Composition Database Fineli:  810 mg/kg.

^d^ Internal method, GC FID; MPL for propionates (E 280–283) in EU: 2,000 mg/kg [18].

### Epidemiological analysis

In the univariable analysis, flour tortillas and the vegetable filling had the highest RRs (Table 3). In the multivariable analysis, we included exposures with a p value < 0.2 (tortilla, vegetable filling, water, pickled red onion, milk and tomato). Eating flour tortillas (aOR = 3.3; 95% CI: 1.4–7.4; p = 0.004) and the vegetable filling (aOR = 1.5; 95% CI: 1.1–2.1; p = 0.005) were associated with developing symptoms.

**Table 3:** Results of univariable and multivariable models in an investigation of a food-borne outbreak linked to consumption of tortillas at a school lunch, Eastern Finland, August 2023 (n = 3,190 respondents)

Food item	Univariable model									Multivariable model		
	Exposed			Unexposed								
	Total	Cases	AR (%)	Total	Cases	AR (%)	RR	95% CI	p value	Adjusted OR^a^	95% CI	p value
Vegetable filling	2,066	557	27	810	142	18	1.54	1.30–1.81	0.000	1.54	1.14–2.08	0.005
Flour tortilla	2,955	710	24	120	8	7	3.60	1.84–7.06	0.000	3.28	1.45–7.42	0.004
Water	1,684	459	27	901	202	22	1.22	1.05–1.40	0.007	1.29	1.01–1.67	0.049
Pickled red onion	568	122	21	2,000	516	26	0.83	0.70–0.99	0.035	0.70	0.44–1.12	0.136
Milk	1,163	278	24	1,481	391	26	0.90	0.79–1.03	0.139	0.99	0.86–1.13	0.842
Tomato	1,655	398	24	990	262	26	0.91	0.79–1.04	0.165	0.90	0.75–1.09	0.281
Margarine	682	180	26	1,775	449	25	1.04	0.90–1.21	0.577	Not included		
Iceberg lettuce	2,164	549	25	467	117	25	1.01	0.85–1.20	0.887			
Crispbread	656	167	25	1,819	468	26	0.99	0.85–1.15	0.892			

AR: attack rate; CI: confidence interval; OR: odds ratio; RR: relative risk.

^a^ Adjusted for mutual effects of the food exposures tested.

Of the 721 cases, 167 (23%) mentioned a bad or strange taste, 24 (3%) a bad or strange odour in tortillas or tortilla portions, and 130 (18%) said that the food tasted of soap or detergent.

### National food- and waterborne outbreaks 2010–2022

Between 2010 and 2022, 640 food- or waterborne outbreaks were reported in Finland. Of these, 602 (94%) were categorised as food-borne and 38 (6%) as waterborne. A chemical agent was considered the causative agent in 20 (3%) outbreaks: 18 were food-borne and two waterborne. Of the estimated 11,922 cases in all food-borne outbreaks, 153 (1.3%) were linked to outbreaks caused by chemical agents (Table 4). Of the 2,424 cases in all waterborne outbreaks, six (0.2%) were linked to outbreaks caused by chemical agents.

**Table 4:** Food- and waterborne outbreaks linked to chemical agents and reported to the Food- and Waterborne Outbreak Registry, Finland, 2010–2022 (n = 20)

Chemical agent	Year	Outbreaks (n = 20)	Patients (n = 159)	Vehicle
Food-borne				
Histamine	2010	1	3	Tuna
	2012	1	28	Fish fillet
	2013	3	27	Tuna, mixed food
	2014	1	23	Escolar
	2018	1	2	Tuna
	2019	1	3	Tuna
	2021	1	9	Mackerel fillet
Copper	2011	1	5	Juice heated in a kettle
	2012	1	6	Mulled wine heated in a kettle
	2013	1	10	Mulled wine heated in a kettle
Wax esters	2010	1	5	Escolar
Sodium glutamate	2010	1	4	Chicken dish at a restaurant
Tropane alkaloids	2013	1	10	Seed pods of *Datura stramonium* in packaged frozen vegetables
Lectin	2014	1	12	Chickpea paste
Sodium nitrite	2017	1	4	Sausage patty
Unidentified biogenic amine	2022	1	2	Tuna
Waterborne				
District heating chemicals	2013	1	4	Drinking water
Lye oversupply	2013	1	2	Drinking water
Total		20	159	NA

NA: not applicable.

## Outbreak control measures

Immediately after the outbreak identification, the MEHA informed local healthcare units to prepare for possible cases. The parents of the students attending these schools were informed via the school’s electronic information system on the afternoon of 16 August.

The environmental health manager was responsible for communication in cooperation with the municipality's communication manager. Eleven press releases were issued between 16 August and 15 November 2023: the first one 3.5 h after the first information about the outbreak (Figure). The first four press releases were to inform the public about the outbreak and to invite exposed people to contact the MEHA. In seven press releases, the investigation results were communicated. The municipality’s communications unit followed the social media discussion but did not participate in it.

The wholesaler that supplied the tortillas and most of the filling components to the schools discontinued the sale of the identified product batches on 18 August (Figure). As the investigation progressed and the suspicion narrowed to tortillas, the filling components were released for sale. Although the suspicion regarding the tortillas was later limited to one batch and a packaging time window (13:12–16:33), the operator destroyed both batches.

An Alert and Cooperation Network (ACN) notification regarding the tortillas was issued by the FFA on 31 August (Figure). The notification was updated six times between 8 September and 16 November as the investigation progressed. The manufacturer was unable to identify any cause in the production process that could explain the abnormal concentration of calcium propionate.

## Discussion

We describe an outbreak involving over 700 students and school staff associated with consumption of flour tortillas served at school lunch in one municipality in Finland in 2023. Our investigation indicated that an excessive amount of the preservative calcium propionate in the tortillas was the likely cause of the outbreak. The likelihood of having gastrointestinal symptoms was three times higher for those eating tortillas than for those who had not eaten them. Albeit few samples tested, we measured nine times higher concentrations of calcium in tortillas packed within 9 min in the afternoon compared with tortillas from the same lot and packaged later that day. Calcium propionate was analysed in three tortilla samples with high calcium content, and 10 times higher concentrations were measured compared with samples from two tortillas produced later. The concentrations exceeded the highest permitted use amount of the additive in tortillas in the EU (2,000 mg/kg) [18]. An unusual vinegar-like odour could have been due to propionic acid, since it is known to have a pungent and rancid odour [19]. The food control authorities in the country where the tortillas were produced were contacted via the ACN to obtain information on any possible explanations in the production process or in the manufacturing of the implicated tortilla batch that could account for the abnormal calcium propionate concentration. The manufacturer did not identify any cause in the production process for the elevated concentrations of calcium propionate.

Several outbreaks of gastrointestinal illness associated with flour tortillas were reported in the US between 1997 and 2004 [20-22]. Most of them occurred in schools and were characterised by short incubation periods and durations of illness. In the outbreaks, no aetiological agent was identified, but symptoms suggested either a biotoxin or a chemical agent. Elevated calcium propionate concentrations were measured in tortillas in three of these outbreaks [22]. Like in our investigation, high calcium propionate concentrations could have been the cause of at least some of the previous outbreaks in the US. After the US outbreaks in 2003–2004, the manufacturer changed the tortilla recipe and reduced the amount of calcium propionate used in its product [21], which may explain why similar outbreaks were no longer reported in the US.

Calcium propionate (E 282) is an organic salt of propionic acid [23] commonly used in bakery products to inhibit moulds and prolong shelf life. It is authorised as a food additive in the EU [10], and its specific purity criteria have been defined in the EU legislation [11]. In 2016, tortillas were added to the category ‘breads and rolls’ in the appendix of the EU regulation as calcium propionate may be used in these food products as a preservative to extend shelf life [18]. Even though calcium propionate is not classified as an irritant, propionic acid is known to be corrosive to mucous membranes [24]. Large differences exist in the tolerance to propionic acid between different animal species. The EFSA Panel on Food Additives and Nutrient Sources Added to Food re-evaluated the safety of propionates in 2014 and concluded that the available toxicity data did not allow for the allocation of an acceptable daily intake value [23]. Also, the US Food and Drug Administration (FDA) considered calcium propionate as safe to be used in foods [25]. Our investigation suggests that the ingestion of large amounts of calcium propionate can cause gastrointestinal irritation.

Food-borne outbreaks with large numbers of cases, sudden-onset symptoms and chemical agent as a likely cause are unusual in Finland. In 2010, sudden-onset symptoms were seen in several outbreaks associated with consumption of raw beetroot [26]. Between 2010 and 2022, chemical agents were considered causing 18 reported food-borne outbreaks with 153 estimated cases. Chemicals are rarely reported causes of outbreaks and complaints about a specific product on the market exceeding toxicity limits are uncommon [14]. A chemical causing a food-borne outbreak may also go unrecognised since identifying the contaminants originating from food production, food processing or packaging can be challenging [27]. Our investigation reminds us of chemical agents as potential causes of food-borne outbreaks and underscores the need for strengthened surveillance in Europe to identify them.

In addition to calcium propionate, we investigated other possible causes. Tests for toxins were selected based on the symptoms and the acute onset, and the availability of analytical methods was also considered. We did not find evidence for *B. cereus* emetic toxin and staphylococcal enterotoxins, common in food-borne outbreaks [28,29], based on negative culture and toxin results. We also investigated contaminants such as mycotoxins, moulds and metals (cadmium, copper, iron, zinc), with all results within acceptable limits. Because of reports of a soapy taste in the tortillas, we considered detergent contamination as a possible cause. These substances can cause gastrointestinal symptoms [30], but reliable methods for detecting them in food are lacking [21]. Phosphorus levels, a possible indicator of industrial detergents, were within the normal range, and the amount required to cause symptoms would likely have made the food unpalatable [21]. Rancid oil has previously been associated with outbreaks [31,32]. However, rancidity in dry foods requires extended exposure to oxygen [33], and since the tortilla packages were opened just before serving, we considered this unlikely.

The vegetable filling, containing Quorn (*Fusarium venenatum*) and red kidney beans, was also investigated. Quorn and kidney beans have been linked to mild gastrointestinal symptoms [34-36], but lectin levels were below the detection limit, and the short incubation period did not support this cause [36]. Tracing of the ingredients further supported that the filling and salad components were not the source. Based on the clinical picture and possible intermediary foods, testing of nitrites was not considered appropriate.

There were limitations in this study. The causal relationship between calcium propionate consumption and the illness was not proven, since no samples were taken from the cases. To promote this, municipal and regional food-borne outbreak preparedness plans could emphasise the collection of patient samples. For example, vomit samples were used for toxin analysis in previous sudden-onset outbreaks in Finland [26]. We recommend obtaining clinical samples from 5–10 symptomatic individuals in future outbreaks. As in many observational studies, bias and confounding cannot be ruled out [37]. Although the questionnaire was distributed promptly, the respondents may have discussed the taste and suspected the tortillas beforehand, possibly influencing their responses.

The outbreak also revealed gaps in communication. The central kitchen, not the schools, reported the symptomatic students to the authorities after being contacted by the satellite kitchens about the ill students. The schools lacked an internal reporting system, and staff considered symptom information confidential and did not share it. We recommend clearer protocols for schools to report sudden illness and interrupt meal service when needed. Early contact with public health authorities enables timely response and sample collection.

Despite these challenges, the MEHA responded rapidly. Food samples and packaging were quickly secured, and efficient coordination between kitchens enabled same day traceback investigations. Food safety management practices of kitchens and distributors further supported the investigation through accurate records and retained food samples.

## Conclusion

An excessive amount of preservative calcium propionate in flour tortillas was the likely cause of gastrointestinal illness in ca 700 people during or after school meals in one Finnish municipality. Our findings remind us of chemical agents as causes of food-borne outbreaks and the need for enhanced surveillance in Europe. Schools should be better prepared for sudden-onset food-borne outbreaks, and guidelines to interrupt school meals for safety reasons should exist.

## Data Availability

The data analysed in this study cannot be shared due to the European General Data Protection Regulation.

## Supplementary Data

251000 Supplementary Material
