# Supplementary material for: Calcium propionate in tortillas – a likely cause of a large outbreak of acute gastrointestinal illness, Finland, 2023
Source: Euro Surveill. 2026 Mar 19;31(11):2500507. doi: 10.2807/1560-7917.ES.2026.31.11.2600185 (PMC13235651; doi:10.2807/1560-7917.ES.2026.31.11.2600185)
Supplement: Supplementary Material [file 25-00507_VARO_Supplement.pdf]

| Analyte | Sample food | Number of samples tested | Methods | Results |
|---|---|---|---|---|
| Trace metals (arsenic, cadmium, chrome, copper, nickel, lead, calcium [a], iron, potassium, magnesium, manganese, sodium, selenium, zinc) and phosphorus | Flour tortillas | 7 / 9[a] | ICP-MS analysis after sample digestion in microwave oven using nitric acid, internal method;<br>ICP-OES analysis after sample digestion in microwave oven using nitric acid and hydrogen peroxide, internal method | Calcium: 5400-5500 mg/kg;<br>Other metals and phosphorus: within expected range |
| Coagulase positive staphylococci | Flour tortillas, filling components (canned kidney beans, frozen Quorn® mince, frozen onion, frozen paprika, canned tomato paste) | 13 | NMKL 66:2009, modified | <100 cfu/g |
| Bacillus cereus | Flour tortillas, filling components (canned kidney beans, frozen Quorn® mince, frozen onion, frozen paprika, canned tomato paste) | 13 | NMKL 67/2021 | <100 cfu/g |
| Aerobic bacteria, 30°C 48 h | Flour tortillas | 8 | NMKL 189/2017 | <100 cfu/g |
| Aerobic sporulating bacteria, 30°C | Flour tortillas | 7 | NMKL 189/2017 | <100 cfu/g |
| Anaerobic sporulating bacteria, 30°C | Flour tortillas | 7 | NMKL 189/2017 | <100 cfu/g |
| Enterobacterales | Flour tortillas | 8 | NMKL 144/2005 | <10 cfu/g |
| Staphylococcal enterotoxins | Flour tortillas, vegetable filling, filling components (canned kidney beans, frozen Quorn® mince, frozen onion, frozen paprika, canned tomato paste), pickled red onion, tomato, iceberg lettuce | 17 | SFS-EN ISO 19020:2017 | Nondetectable, limit of detection 0,007 ng/g (SEA)* |
| Cereulide | Flour tortillas, vegetable filling, filling components (canned kidney beans, frozen Quorn® mince, frozen onion, frozen paprika, canned tomato paste), pickled red onion, tomato, iceberg lettuce | 17 | LC-MS/MS, ISO 18465:2017 | Nondetectable,<br>limit of detection 1.0 µg/kg |
| Mycotoxins (Deoxynivalenol, DON-3G, Fusarenon-X, 15-AcDON, 3-AcDON, Diacetoxyschirpenol, Neosolaniol, Nivalenol, HT-2, T-2, Ochratoxin, Fumonisin B2, Fumonisin B1, Zearalenone) | Flour tortillas | 7 | LC-MS/MS analysis after extraction with acetonitrile/water/ formic acid solution , internal method | Nondetectable,<br>limit of detection 0.45-28 µg/kg |
| Moulds | Flour tortillas | 8 | NMKL 98:2005, modified;<br>OGYE agar, 25°C 5-7 days | <100 cfu/g |
| Lectin | Canned kidney beans | 1 | immunoagglutination of erythrocytes, internal method | <0,05 mg/kg |

## First phase laboratory testing of food samples in a food-borne outbreak caused by calcium propionate in Finland, 2023

*As consolidated data were only available for SEA, it was decided to use this value also for the other toxin types SEB to SEE.

This supplementary material is hosted by Eurosurveillance as supporting information alongside the article Calcium propionate in tortillas – a likely cause of a large outbreak of acute gastrointestinal illness, Finland, 2023, on behalf of the authors, who remain responsible for the accuracy and appropriateness of the content. The same standards for ethics, copyright, attributions and permissions as for the article apply. Supplements are not edited by Eurosurveillance and the journal is not responsible for the maintenance of any links or email addresses provided therein.
